# Leukocyte telomere length and risk of gastric cardia adenocarcinoma

**DOI:** 10.1038/s41598-018-32954-6

**Published:** 2018-10-01

**Authors:** Yang Liu, Tianshui Lei, Nasha Zhang, Yan Zheng, Peisi Kou, Shuheng Shang, Ming Yang

**Affiliations:** 1 School of Medicine and Life Sciences, University of Jinan-Shandong Academy of Medical Sciences, Jinan, Shandong Province China; 2 Shandong Provincial Key Laboratory of Radiation Oncology, Cancer Research Center, Shandong Cancer Hospital affiliated to Shandong University, Shandong Academy of Medical Sciences, Jinan, Shandong Province China; 3 Department of Radiation Oncology, Shandong Cancer Hospital affiliated to Shandong University, Shandong Academy of Medical Sciences, Jinan, Shandong Province China

## Abstract

As a chromosome stabilizer, telomeres play an essential part in maintaining the stability and integrity of human genome. Shortened telomeres have been associated with the development of cancers but it is still largely unclear whether leukocyte telomere length contributes to predisposition of gastric cardia adenocarcinoma (GCA). We conducted a case-control study consisting of 524 GCA cases and 510 controls to assess the association between telomere length in peripheral blood leukocytes and GCA risk in a Chinese Han population. The GCA patients had significantly overall shorter relative leukocyte telomere length (RTL) (median ± SD: 1.10 ± 0.54) when compared with the controls (1.24 ± 0.58). Individuals with the shortest quartile of RTL performed a doubled GCA risk (OR = 2.18; 95% CI = 1.47–3.22, *P* = 9.90 × 10^−5^) when compared with those with the highest quartile. We also found that telomere shortening and smoking have a significantly synergistic effect in intensifying risk of GCA (OR = 7.03, 95% CI = 4.55–10.86, *P* = 1.43 × 10^−18^). These findings indicate that short RTL contributes to increased susceptibility of gastric cardia adenocarcinoma and might be a promising marker to identify high-risk individuals combined with lifestyle risk factors.

## Introduction

There are inconsistent results in studies examining relative leukocyte telomere length (RTL) in gastric cancer (GC)^1–5^. GC cases had significantly shorter RTL than controls and GC risk doubled [odds ratio (OR) = 2.04; 95% confidence interval (95% CI) = 1.33–3.13] among subjects in the shortest compared with the highest quartile of telomere length in a high-risk Polish population^1^. Similarly, we assessed the association of RTL with risk of GC or esophageal squamous cell carcinoma (ESCC) in a Chinese Han population^2^ and found consistent results as the previous data in Polish. Four-fold increased GC risk (95% CI = 2.78–6.05, *P* = 1.10 × 10^−12^) among individuals in the shortest quartile of telomere length was found compared with the highest quartile^2^. A meta-analysis provided strong evidences that short surrogate tissue (e.g., blood cells) RTL is associated with GC^3^. Contrastingly, Du *et al*. observed a U-shaped association between telomere length and GC risk (*P* < 0.001), with ORs (95% CIs) being 3.81 (2.82–5.13), 1.65 (1.21–2.26), 1.28 (0.93–1.77) and 1.78 (1.30–2.44) for individuals in the first (the shortest), second, third and fifth (the longest) quintile as compared with those in the fourth quintile as reference group in a Southern Chinese population^4^. In line with this, Wang *et al*. found a U-shaped association between telomere length and risk of gastric adenocarcinoma in the Singapore Chinese Health Study^5^. However, the role of telomere length on development of gastric cardia adenocarcinoma (GCA) is largely unclear.

GCA is one of the most common and deadly malignancies in the world and China alone accounts for more than half of GCA cases^6–8^. Unlike GC at other sites, GCA is a special type of GC having its own epidemiological characteristics, etiology, pathogenesis and clinical manifestations^9^. However, GCA and esophageal cancer may have similar patterns of age and sex distribution and morphology^9^ and share common environmental risk factors such as cigarette smoking, heavy alcohol consumption, dietary carcinogen exposure, low-intake of fruits and vegetables and gastroesophageal reflux disease^7,10–13^. Considering there is no reports on impacts of telomere length on GCA risk, we conducted a case-control study consisting of 524 GCA cases and 510 age and sex matched controls in a Northern Chinese Han population to evaluate the role of RTL in GCA.

## Materials and Methods

### Study subjects

All GCA cases and controls included in this study are Chinese Han population and have no genetic relationship. A total of 524 GCA cases were consecutively recruited between August 2012 and August 2017 at Shandong Cancer Hospital affiliated to Shandong University. All patients were histologically confirmed as GCA. Exclusion criteria was that patients with a second primary tumor or the primary tumor outside of gastric cardia. A total of 510 cancer-free control subjects were randomly selected from a pool of 6000 individuals from a comprehensive physical examination conducted in Jinan city and the surrounding areas during the same time period as the patients were collected. All controls were frequency matched to cases based on age and sex. Individuals were considered as smokers, if they smoked one cigarette per day for over one year. Subjects who drank at least once per week were considered as alcohol drinkers. At recruitment, all subjects signed the informed consents. This study was approved by the institutional Review Board of Shandong Cancer Hospital affiliated to Shandong University.

### Measurement of relative telomere length

Telomere length was measured in genomic DNA of peripheral blood leukocytes by SYBR Green quantitative polymerase chain reaction (qPCR) protocol as reported in previous studies^14,15^. The qPCR primers of telomere were TEL1 (5′-GGTTTTTGA[GGGTGA]_4_GGGT-3′) and TEL2 (5′- TCCCGACTAT[CCCTAT]_4_CCCTA-3′). The qPCR primers of the single-copy gene (β2-globulin) were HBG1 (5′-GCTTCTGACACAACTGTGTTCACTAGC-3′) and HBG2 (5′-CACCAACTTCATCCACGTTCACC-3′). All samples reactions were performed twice and the mean of two measurements was used in the statistical analyses. The laboratory operators were blinded to both case and control status. RTL was determined via Cawthon’s formula^15^. In brief, the reference DNA was mixed DNA from 50 individuals who were randomly selected from the 510 control subjects in the current study. RTL in peripheral blood leukocytes was calculated by the ratio of the copy number of telomere repeat (T) to the copy number of single copy gene (S) and represented as T/S ratio in individual sample.

### Statistical analyses

Pearson χ^2^ test was used to evaluate differences of the demographic data distribution (age and sex) and GCA risk factors (smoking status and drinking status) between cases and controls. The correlation between RTL and age was examined by generalized linear models. Mann-Whitney U test was employed to test continuous variables such as RTL between cases and controls. Student *t*-test was employed to test continuous variables such as RTL between cases and controls. RTL was also analyzed as a categorial variable and was categorized into four groups according to its quartile distribution in controls. The logistic regression model was utilized to calculate the OR and 95% CI in order to evaluate the association between RTL and GCA risk in each quartile. In addition, we also carried out a stratified analysis to test RTL associated with GCA risk among different subgroups stratified by age, sex, smoking status, and drinking status. Analyses were all adjusted for age, sex, cigarette smoking status and drinking consumption as appropriate. All statistical tests were two-sided and a *P* value of less than 0.05 was considered statistical significance. All analyses were performed using SPSS 16.0 (SPSS Inc.).

## Results

### Subject characteristics and risk factors

**Figure 1:**
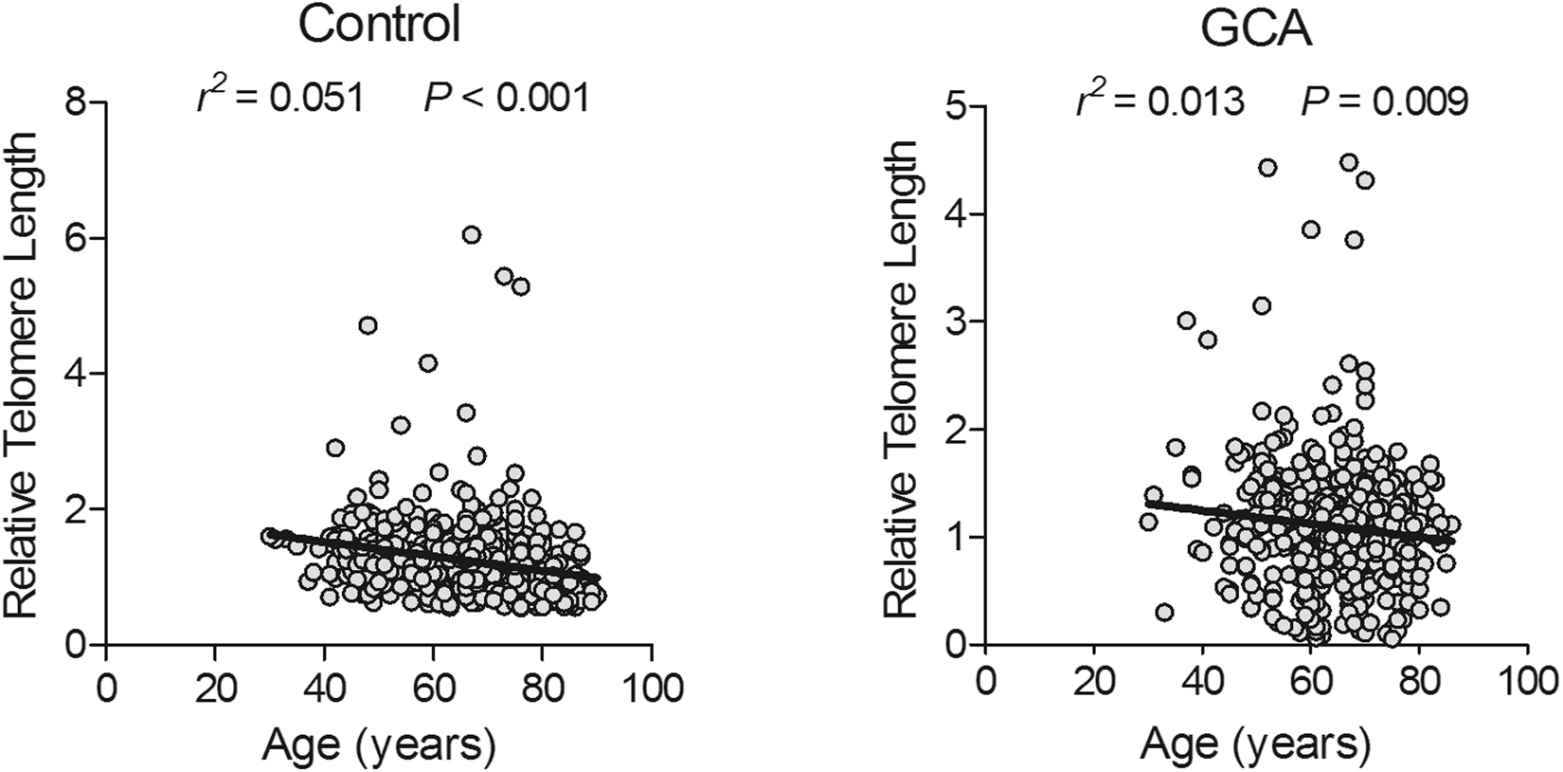
Correlation between leukocyte relative telomere length and age in different groups. Relative telomere length in the control group and the GCA group.

As shown in Table 1, there were 524 GCA cases and 510 controls recruited in this study. No significant statistical differences were found in the distributions of median age and sex between GCA patients and controls (all *P* > 0.05), as age and sex were adequately matched. However, smokers and drinkers accounted for a higher proportion among GCA patients compared with those in controls (smoking: 46.37% vs. 21.76%, *P* < 0.0001; drinking: 40.84% vs. 32.75%, *P* = 0.002). Additionally, when compared with the controls, the GCA patients had significantly shorter RTL (median ± SD = 1.10 ± 0.54 vs. 1.24 ± 0.58, *P* < 0.001). As expected, RTL of peripheral blood leukocytes shortened significantly with increasing age at recruitment in both controls and GCA cases (controls: *r*^2^ = 0.051, *P* < 0.001; GCA: *r*^2^ = 0.013, *P* = 0.009) (Fig. 1).

**Table 1:** Distribution of selected characteristics among GCA cases and controls.

Variables	Controls	Cases	*P* value
	N = 510 (%)	N = 524 (%)	
Age at diagnosis (years)			0.834
<60	141 (27.65)	141 (26.91)	
≥60	369 (72.35)	383 (73.09)	
Sex			0.154
Male	435 (85.29)	429 (81.87)	
Female	75 (14.71)	95 (18.13)	
Smoking status			2.69 × 10^−14^
No	399 (78.24)	269 (51.34)	
Yes	111 (21.76)	243 (46.37)	
No data		12 (2.29)	
Drinking status			0.002
No	343 (67.25)	294 (56.11)	
Yes	167 (32.75)	214 (40.84)	
No data		16 (3.05)	
Relative telomere length			3.34 × 10^−5^
Mean ± SD	1.24 ± 0.58	1.10 ± 0.54	

Abbreviations: GCA, gastric cardia adenocarcinoma; OR, odds ratio; CI, confidence interval; SD, standard deviation.

*P* values of GAC case-control set were calculated by Pearson chi-square tests for age, sex, smoking and drinking status or Mann-Whitney U test for relative telomere length.

### Telomere length and GCA risk

Unconditional logistic regression analysis was used to evaluate the association between RTL and risk of GCA (Table 2). We first dichotomized RTL into the long telomere group and the short telomere group based on the median telomere length of controls. With an adjustment for age, sex, smoking status and drinking status, we found a significantly elevated GCA risk (OR = 1.71; 95% CI = 1.31–2.24; *P* = 9.05 × 10^−5^) among patients with short RTL. We further categorized subjects into four groups according to the RTL quartile distribution in controls (Table 2). Quartiles analyses of RTL showed that individuals with the first quartile of the shortest RTL (RTL < 0.898) had a doubled GCA risk (OR = 2.18; 95% CI = 1.47–3.22; *P* = 9.90 × 10^−5^) when compared with those in the fourth quartile of the longest RTL (RTL ≥ 1.448). In addition, when compared with the lowest risk individuals in the fourth quartile, the adjusted OR (95% CI) of RTL among cases in the second (RTL = 0.898–1.179) and third (RTL = 1.180–1.447) quartiles were 1.49 (95% CI = 1.00–2.22) and 2.07 (95% CI = 1.40–3.04) respectively, indicating a significant dose-response relationship between telomere length shortening and increased GCA risk.

**Table 2:** Risk estimates for leukocyte relative telomere length and GCA.

Variables	Relative Telomere Length	Controls, N (%)	Cases, N (%)	OR (95% CI)^a^	*P* value^a^
Overall telomere length					
Long	≥1.180	255 (50.00)	201 (38.36)	1.00	
Short	<1.180	255 (50.00)	323 (61.64)	1.71 (1.31–2.24)	9.05 × 10^−5^
Quartile					
75–100%	≥1.448	127 (24.90)	90 (17.18)	1.00	
50–75%	1.180–1.447	128 (25.10)	111 (21.18)	1.49 (1.00–2.22)	0.052
25–50%	0.898–1.179	128 (25.10)	160 (30.53)	2.07 (1.40–3.04)	2.33 × 10^−4^
0–25%	<0.898	127 (24.90)	163 (31.11)	2.18 (1.47–3.22)	9.90 × 10^−5^
				*P*_trend_^b^	3.38 × 10^−5^

Abbreviations: GCA, gastric cardia adenocarcinoma; OR, odds ratio; CI, confidence interval; SD, standard deviation.

^a^Data were calculated by logistic regression with adjustments for age, sex, smoking and drinking status.

^b^Test for trend of odds was two-sided and based on likelihood ratio test assuming a multiplicative model.

When stratified by host characteristics, the similar association between short telomere length and elevated GCA risk were observed in each stratum (Table 3). Interestingly, short RTL was associated with a higher risk of GCA among men (OR = 1.43, 95% CI = 1.07–1.92), but not in females (OR = 1.17, 95% CI = 0.59–2.36). Moreover, short telomere length tended to be more pronouncedly associated with increased risk of GCA among persons with high-risk profile such as cigarette smoking (OR = 2.21; 95% CI = 1.35–3.64) and alcohol drinking (OR = 2.18; 95% CI = 1.33–3.60). In contrast, the ORs for GCA risk in individuals with short RTL were 1.59 (95% CI = 1.14–2.22) among current nonsmokers and 1.58 (95% CI = 1.14–2.20) for nondrinkers, respectively.

**Table 3:** Associations between relative telomere length in peripheral blood leukocytes and GCA risk stratified by age, sex, smoking, and drinking status.

	RTL	Controls		Cases		OR (95% CI)	*P* value^a^
		N	%	N	%		
Age (years)							
<60	Long	94	66.67	66	46.81	1.00	
	Short	47	33.33	75	53.19	2.14 (1.27–3.60)	0.004
≥60	Long	161	43.63	135	35.25	1.00	
	Short	208	56.37	248	64.75	1.38 (1.01–1.88)	0.042
	*P* value^b^	4.54 × 10^−6^		0.020			
Sex							
Male	Long	216	49.66	192	42.57	1.00	
	Short	219	50.34	259	57.43	1.43 (1.07–1.92)	0.016
Female	Long	39	52.00	36	49.32	1.00	
	Short	36	48.00	37	50.68	1.17 (0.59–2.36)	0.652
	*P* value^b^	0.803		0.131			
Smoking status							
No	Long	203	50.88	112	41.64	1.00	
	Short	196	49.12	157	58.36	1.59 (1.14–2.22)	0.007
Yes	Long	52	46.85	83	34.16	1.00	
	Short	59	53.15	160	65.84	2.21 (1.35–3.64)	0.002
	*P* value^b^	0.520		0.084			
Drinking status							
No	Long	162	47.23	116	39.46	1.00	
	Short	181	52.77	178	60.54	1.58 (1.14–2.20)	0.007
Yes	Long	93	55.69	77	35.98	1.00	
	Short	74	44.31	137	64.02	2.18 (1.33–3.60)	0.002
	*P* value^b^	0.089		0.459			

Abbreviations: GCA, gastric cardia adenocarcinoma; RTL, Relative Telomere length; OR, odds ratio; CI, confidence interval; SD, standard deviation.

^a^*P* values and ORs were adjusted for age, sex, smoking and drinking status with a logistic regression model.

^b^*P* values were calculated by Pearson chi-square tests for age, sex, smoking and drinking status.

### Interaction of RTL and cigarette smoking on GCA risk

Given that cigarette smoking is a risk factor of GCA, we combined leukocyte RTL and cigarette smoking status to evaluate the cumulative effects of these two risk factors on GCA. As shown in Table 4, although nonsmokers with short RTL had an increased risk of GCA (adjusted OR = 1.65; 95% CI = 1.19–2.28) than nonsmokers having long RTL, the group of smokers with short RTL had highest GCA risk, with the OR being 7.03 (95% CI = 4.55–10.86, *P* = 1.43 × 10^−18^), even higher than the product of the OR for smokers with long RTL (adjusted OR = 3.79; 95% CI = 2.36–6.09) and the OR for nonsmokers with short RTL (i.e., 1.65 × 3.79 = 6.25). These results showed that smoking and short RTL in peripheral blood leukocytes had a synergistic effect on risk of GCA (Table 4).

**Table 4:** Interaction of leukocyte telomere length and cigarette smoking on GCA risk.

RTL^a^	Smoking status	Controls, N (%)	Case, N (%)	OR (95% CI)^b^	*P* value^b^
Long	No	203 (39.80)	112 (21.88)	1.00	—
Short	No	196 (38.43)	157 (30.66)	1.65 (1.19–2.28)	0.003
Long	Yes	52 (10.20)	83 (16.21)	3.79 (2.36–6.09)	3.35 × 10^−8^
Short	Yes	59 (11.57)	160 (31.25)	7.03 (4.55–10.86)	1.43 × 10^−18^

Abbreviations: GCA, gastric cardia adenocarcinoma; RTL, Relative Telomere length; OR, odds ratio; CI, confidence interval; SD, standard deviation.

^a^The long and short groups were dichotomized at the median telomere length in the controls.

^b^Data were calculated by logistic regression with adjustment for age, sex and drinking status.

*P*_interaction_ = 1.27 × 10^−13^ (*P* values for gene-smoking interaction).

## Discussion

In the current study, we investigated whether telomere length in peripheral blood leukocytes contributes to GCA predisposition. Our found that GCA patients exhibit a significantly shorter leukocyte RTL than controls and an increased risk of developing GCA was associated with progressively telomere length shortening. In addition, a cumulative effect between short telomeres and cigarette smoking on GCA risk was also found.

Function as a chromosome protector, telomeres play an important role in the maintenance of genomic stability^16–21^. Telomeres are DNA-protein structures, consisting of “TTAGGG” short repetitive sequences and terminal protein complex. The progressive shortening of the telomere induces chromosomal instability of normal somatic cells^20^. In fact, telomere shortening is associated with a variety of aging-related diseases including cancers^21^. However, there are conflicting results of the relationship between telomere length and the risk of incidence of cancers^21^. In a meta-analysis of prospective and retrospective case-control studies, Wentzensen *et al*. found that short telomeres were associated with an increased risk to developing several cancers, such as esophageal cancer, GC, bladder cancer, and renal cancer^3^. In a case-control study of GC consisting of 396 patients and 378 controls, Liu *et al*. measured lymphocyte average telomere length and observed that short telomere length was significantly associated with an increased GC risk (OR = 2.14, 95% CI = 1.52–2.93)^22^. Similarly, Xing *et al*. designed a case-control study consisting of 94 esophageal carcinoma patients and 94 matched controls and reported that the overall lymphocyte telomere length in patients were significantly shorter than controls (*P* = 0.004). Moreover, subjects with shorter overall telomere length had a significant elevated risk in the development of esophageal cancer (OR = 2.52, 95% CI = 1.29–4.94)^23^. Our findings that short telomere length was associated with an elevated GCA risk were consistent with its role in the risk to developing esophageal cancer and GC. However, other studies showed inconsistent results. An obvious U-shaped association pattern between telomere length and GC risk has been observed by Du *et al*. in a case-control study consisting of 1136 GC cases and 1012 controls, implying that both the lowest and the highest quintiles of telomere length were significantly correlated with higher GC risk as compared with the reference group (the fourth quintile)^4^. Svenson *et al*. found that longer telomere length was significantly associated with an increased risk of breast cancer by analyzing telomere length among 256 patients and 446 matched health controls^24^. Interestingly, Sanchez-Espiridion *et al*. indicated that the association between telomere length and lung cancer risk was histologically dependent while lung adenocarcinoma patients had longer telomere length, whereas shorter telomere was dominant among cases with lung squamous cell carcinoma^25^. Given the inconsistent results of the association between telomere length and the susceptibility of cancers, further studies are warranted to assess the possibility of telomere length as a biomarker to evaluate the risk of cancers.

Smoking is one of major environmental risk factors of GCA. Interestingly, tobacco smoking can accelerate the process of telomere shortening^26^. For instance, McGrath *et al*. observed that healthy individuals who smoked had significantly shorter telomeres than nonsmokers^27^. A dose-effect association has been observed between the cumulative long-life exposure to tobacco smoking and telomere length^28^. These phenomena can be attributed to the oxidative stress induced by smoking. Indeed, cigarette smoke increases the oxidative stress not only by directly acting as a source of reactive oxygen species (ROS) but also by activating inflammatory cells and endogenous generation of ROS consequently^29^. Acting as a significant modulator of telomere attrition, oxidative stress could accelerate telomere shortening and contribute to the process of aging^30,31^. Consistent with these epidemiology data, we observed a significantly synergistic effect between short telomeres and smoking in elevating GCA risk. Individuals in our study who had two risk factors, short telomere length and smoking, exhibited a 7.03-fold elevated risk to develop GCA (*P* = 1.43 × 10^−18^), although short RTL is an independent risk factor for GCA. These results elucidated the hypothesis that short telomere length can serve as a molecular marker to predict and evaluate GCA risk.

In all, this is the first study demonstrating that subjects with short telomeres may have an increased risk of GCA in Chinese population, which provided precious clues for better interpretation the underlying contribution of telomeres to GCA development and implied the potential of short telomeres as a biomarker for assessing individuals with high GCA risk. Future studies, preferably in well-designed prospective studies, may facilitate to confirm our findings.

### Ethical approval and informed consent

All procedures followed were in accordance with the ethical standards of the responsible committee on human experimentation (institutional and national) and in compliance with the Helsinki Declaration of 1964 and later versions. Shandong Cancer Hospital affiliated to Shandong University discloses information to the patients. Participating patients were excluded only when they specified that they were unwilling to participate.

## Electronic supplementary material

Supplementary information
