# Supplementary material for: Leukocyte telomere length and risk of gastric cardia adenocarcinoma
Source: Sci Rep. 2018 Oct 1;8:14584. doi: 10.1038/s41598-018-32954-6 (PMC6167366; doi:10.1038/s41598-018-32954-6)

Leukocyte telomere length and risk of gastric cardia adenocarcinoma

Yang Liu[1,2,3]; Tianshui Lei[1,2]; Nasha Zhang[2]; Yan Zheng[2]; Peisi Kou[2]; Shuheng Shang[2]; Ming Yang[2,*]

[1]School of Medicine and Life Sciences, University of Jinan-Shandong Academy of Medical Sciences, Jinan, Shandong Province, China;
[2]Shandong Provincial Key Laboratory of Radiation Oncology, Cancer Research Center, Shandong Cancer Hospital affiliated to Shandong University, Shandong Academy of Medical Sciences, Jinan, Shandong Province, China;
[3]Department of Radiation Oncology, Shandong Cancer Hospital affiliated to Shandong University, Shandong Academy of Medical Sciences, Jinan, Shandong Province, China.

***Correspondence to:** Ming Yang, PhD, Professor, Shandong Provincial Key Laboratory of Radiation Oncology, Cancer Research Center, Shandong Cancer Hospital affiliated to Shandong University, Shandong Academy of Medical Sciences, Jinan 250117, Shandong Province, China. Tel & Fax: 86531-67626536; E-mail: aaryoung@yeah.net, yangm@sdu.edu.cn.

Supplementary Figure 1

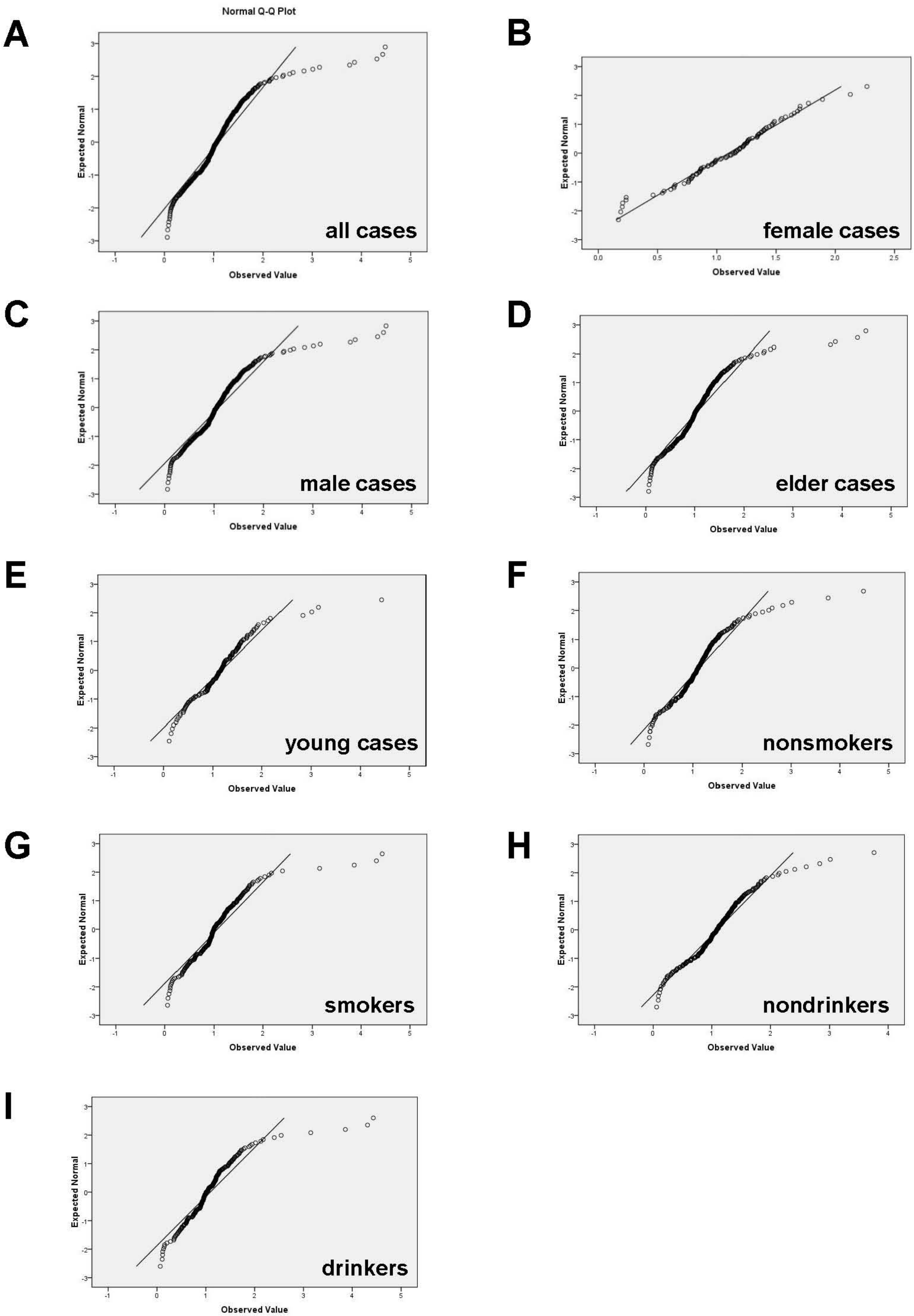

Supplementary Figure 2

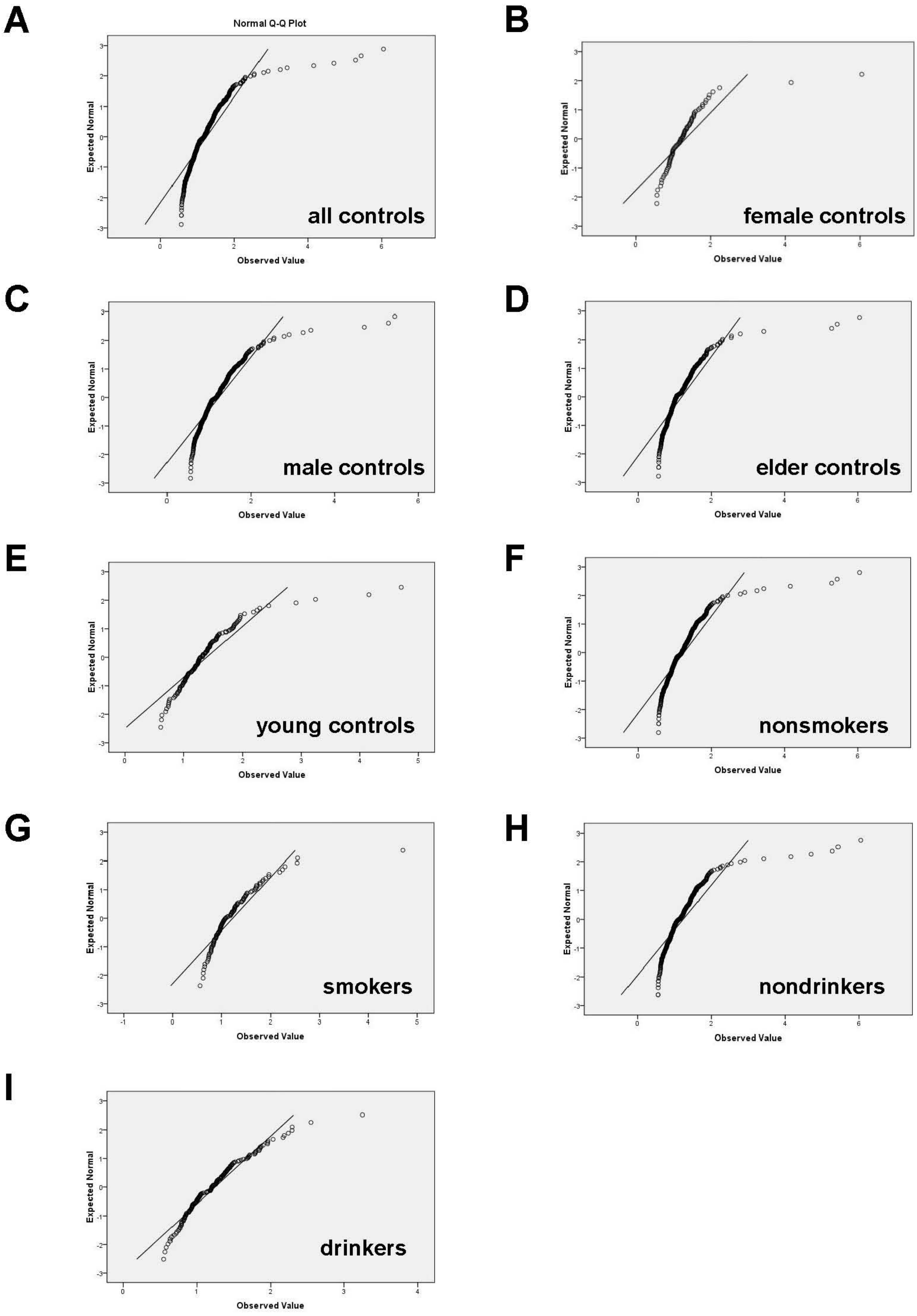

Supplement: Supplementary file 1 — Supplementary information [file 41598_2018_32954_MOESM1_ESM.pdf]
